# G-CSF and GM-CSF Modify Neutrophil Functions at Concentrations found in Cystic Fibrosis

**DOI:** 10.1038/s41598-019-49419-z

**Published:** 2019-09-10

**Authors:** Stefano Castellani, Susanna D’Oria, Anna Diana, Angela Maria Polizzi, Sante Di Gioia, Maria Addolorata Mariggiò, Lorenzo Guerra, Maria Favia, Angela Vinella, Giuseppina Leonetti, Domenica De Venuto, Crescenzio Gallo, Pasqualina Montemurro, Massimo Conese

**Affiliations:** 1 Department of Medical and Surgical Sciences, University of Foggia, Foggia, Italy; 2 Department of Biomedical Sciences and Human Oncology, Section of General Pathology, University of Bari, Bari, Italy; 3 Department of Biomedical Sciences and Human Oncology, Section of Medical Genetics, University of Bari, Bari, Italy; 4 Department of Biosciences, Biotechnologies and Biopharmaceutics, University of Bari, Bari, Italy; 5 Cystic Fibrosis Regional Center, Department of Biomedical and Human Oncology, Pediatrics Section, U.O. “B. Trambusti”, Policlinico, University of Bari, Bari, Italy; 6 Department of Clinical and Experimental Medicine, University of Foggia, Foggia, Italy

**Keywords:** Cystic fibrosis, Innate immunity

## Abstract

The role of colony stimulating factors (CSFs) in cystic fibrosis (CF) circulating neutrophils has not been thoroughly evaluated, considering that the neutrophil burden of lung inflammation in these subjects is very high. The aim of this study was to assess granulocyte-CSF (G-CSF) and granulocyte-macrophage-CSF (GM-CSF) levels in CF patients in various clinical conditions and how these cytokines impact on activation and priming of neutrophils. G-CSF and GM-CSF levels were measured in sputum and serum samples of stable CF patients (n = 21) and in CF patients with acute exacerbation before and after a course of antibiotic therapy (n = 19). CSFs were tested on non CF neutrophils to investigate their effects on reactive oxygen species (ROS) production, degranulation (CD66b, elastase, lactoferrin, MMP-9), and chemotaxis. At very low concentrations found in CF patients (0.005–0.1 ng/ml), both cytokines inhibited ROS production, while higher concentrations (1–5 ng/ml) exerted a stimulatory effect. While either CSF induced elastase and MMP-9 secretion, lactoferrin levels were increased only by G-CSF. Chemotaxis was inhibited by GM-CSF, but was increased by G-CSF. However, when present together at low concentrations, CSFs increased basal and fMLP-stimulated ROS production and chemotaxis. These results suggest the CSF levels that circulating neutrophils face before extravasating into the lungs of CF patients may enhance their function contributing to the airway damage.

## Introduction

Cystic fibrosis (CF) is a life-threatening condition and the most lethal among the autosomal recessive diseases. It is caused by the absence/dysfunction of CFTR (CF Transmembrane Conductance Regulator) protein, expressed on the plasma membrane of epithelial cells. Although CF is characterized by the dysfunction of many organs, lung disease represents the main cause of morbidity and mortality of these individuals. A neutrophil-dominated inflammatory response is the hallmark of CF lung disease, along with pulmonary infections by opportunistic bacterial pathogens^1^. The CFTR protein is a chloride and bicarbonate channel and also a regulator of other channels, such as the epithelial sodium channel (ENaC). In the CF airways, its absence/dysfunction leads to the lack of chloride and bicarbonate secretion coupled with an exaggerated ENaC activity, increased sodium flux and water absorption from the lumen, thickening and abnormal viscosity of mucus overlaying the epithelium, and eventually abrogation of the mucociliary clearance^2^. The acidification of the airway secretions determines the functional loss of antimicrobial peptides^3^. Bacterial species, such as *Pseudomonas aeruginosa*, took advantage of such alteration of the airway microenvironment by penetrating in the mucus and creating macrocolonies which defend themselves from the attack of the immune system^4,5^. Neutrophils are thereby continuously attracted into the CF airways and activated. The continuous secretion of reactive oxygen species (ROS) is implicated in the damage to the airway epithelial cells, whilst the secretion of granule proteases, such as elastase, is involved in the degradation of extracellular matrix protein of bronchioles, determining bronchiectasis, and thus further worsening the mucociliary clearance. Metalloproteinases (MMPs), in particular MMP-9, also produced by neutrophils, may contribute to matrix breakdown and repair, thus contributing to the damage and remodeling of CF airways^6^. Moreover, neutrophil cytotoxic peptides, granule enzymes, extracellular DNA, and neutrophil extracellular traps (NETs) are associated with increased mucus clogging and lung injury in CF^7^. During NETosis, neutrophils release decondensed chromatin coated with elastase, myeloperoxidase, and other cytotoxic granular proteases, thus perpetuating severe inflammation that characterizes CF lung disease^8,9^. Pro-inflammatory cytokines (IL-1β and TNF-α) and chemoattractants (such as IL-8 and LTB_4_), are elevated in the CF airway milieu, especially during acute exacerbation episodes, and are involved in continuous neutrophils’ extravasation and activation^10–12^. The attraction of neutrophils in the CF airways has been more recently attributed to high burden of the IL-23/IL-17 axis which occurs in patients infected by *P*. *aeruginosa* and undergoing a pulmonary exacerbation^13–15^. IL-17A and IL-17F up-regulate granulopoietic factors and CXC chemokine in airway epithelial cells, thus contributing to the massive neutrophil influx in the CF airways^13^. In the more advanced stages of lung disease, a skewed immune response with a TH2-dominated alveolar inflammation and tolerance towards Gram-negative infections, disfavoring a protective TH1 response, is associated with poor prognosis^16^.

The role of colony stimulating factors (CSFs) in the survival and function of neutrophils is well recognized. In CF, granulocyte-CSF (G-CSF) and granulocyte-macrophage-CSF (GM-CSF) have been found to be elevated in serum and sputum and to correlate negatively with lung disease^17–19^. Mechanistically, GM-CSF is involved in the accumulation of neutrophils in the airways caused by IL-17 and TNF-α, probably via effects on both recruitment and survival of neutrophils^20^. Moreover, GM-CSF has been found to prime neutrophils for NET release by other stimuli, such as LPS or C5a^21^.

However, notwithstanding these findings, the role of CSFs in modifying circulating neutrophils in CF has not been ascertained yet. In the past, we have undertaken a thoughtful study of blood neutrophils’ functions in CF before and after an antibiotic course for a pulmonary exacerbation, finding that oxidative burst and expression of HVCN1, a protein channel involved in the acidification of endosomal vesicles, were modified by the antibiotic therapy^22,23^. In this paper, we have aimed to quantify serum and sputum CSF levels in a cohort of stable CF patients and compare them with those of CF subjects in acute exacerbation and to elucidate whether they are modified by a course of antibiotic therapy. Moreover, based on these results, we have studied the effect of GM-CSF and G-CSF on various neutrophil functions, including oxidative burst, degranulation, and chemotaxis. We chose to carry out this experimental part on non CF neutrophils to avoid biases linked to pre-exposure of CF neutrophils to a systemic inflammatory milieu as has been demonstrated in these patients^24^.

## Results

### Clinical characteristics of CF patients

Clinical parameters of stable and acute patients, before and after a course of antibiotic therapy, are shown in Table 1. Patients in acute exacerbation presented an increase in values concerning systemic inflammation, such as WBC (absolute counts), % neutrophils, and C-reactive protein (CRP) in comparison with stable patients. The antibiotic treatment determined a significant reduction in WBC counts, % neutrophils and CRP levels. FEV_1_, as a parameter of respiratory function, was reduced in patients in acute exacerbations as compared with stable patients and showed a non-significant increase upon antibiotic therapy.

**Table 1:** Clinical parameters of CF subjects involved in this study.

	Stable (n = 21)	Pre-therapy (n = 19)	Post-therapy (n = 19)	P-value
WBC (×10^4^)	7.7 ± 0.5	10.2 ± 0.9	7.4 ± 0.4	**stable vs pre **pre vs. post
Neutrophils (% of WBC)	56.0 ± 2.2	62.5 ± 2.6	54.5 ± 2.0	*pre vs. post
CRP (mg/dl)	16.6 ± 9.9	32.7 ± 9.9	8.7 ± 2.2	**stable vs. pre **pre vs. post
FEV_1_ (% predicted)	66.0 ± 6.1	41.1 ± 2.4	53.3 ± 4.0	***stable vs pre

Data are presented as mean ± SEM. Kruskall-Wallis (with Dunn’s test as post hoc test) and Mann-Whitney U-test: *P < 0.05; **P < 0.01; ***P < 0.0001.

### CSFs levels in sputum and sera of CF patients

Patients in stable state and in acute exacerbation before and after antibiotic therapy were analyzed for G-CSF and GM-CSF levels in sputum and serum specimens and their concentration values are depicted in Supplementary Fig. S1 and reported in Table 2. As shown in Supplementary Fig. S1, serum levels of both cytokines were found at very low concentrations and did not display obvious differences among stable patients and acute patients before and after therapy. However, GM-CSF levels were lower than G-CSF. A very similar pattern was observed for sputum levels and for both cytokines (Supplementary Fig. S1). Data reported in Table 2 show that sputum levels of G-CSF were significantly higher than in serum in stable and acute (before therapy) conditions, whereas GM-CSF levels were similar in sputum and serum. In sputum, levels of both CSFs between stable and acute patients before therapy were similar, while a trend towards decrease was observed in patients after therapy in comparison with pre-therapy values. Interestingly, serum levels of both G-CSF and GM-CSF were lower in acute patients before therapy as compared with stable patients and increased after therapy, although not significantly in both cases. Overall, these data indicate that CSF levels in serum behave oppositely to sputum levels and that an antibiotic course might modify them.

**Table 2:** G-CSF and GM-CSF concentrations in sputum and serum.

	Sputum			Serum		
	Stable	Pre-therapy	Post-therapy	Stable	Pre-therapy	Post-therapy
G-CSF	0.22** (0.13–0.32)	0.21** (0.05–0.36)	0.15 (0.00–0.36)	0.07 (0.02–0.12)	0.04 (0.01–0.09)	0.07 (0.03–0.17)
GM-CSF	0.005 (0.002–0.012)	0.008 (0.003–0.012)	0.004 (0.002–0.014)	0.009 (0.007–0.014)	0.005 (0.003–0.01)	0.01 (0.006–0.01)

G-CSF and GM-CSF levels (ng/ml) are reported as median (interquartile range). Kruskall-Wallis (with Dunn’s test as post hoc test) and Mann-Whitney U-test: **sputum G-CSF stable vs. serum G-CSF stable and sputum G-CSF pre-therapy vs serum G-CSF pre-therapy: P < 0.01.

There was a weak but significant positive correlation observed between sputum GM-CSF levels and WBC prior to therapy in acute patients (r = 0.5694; P < 0.05) and between sputum G-CSF levels and FEV_1_ in stable patients (r = 0.4917; P < 0.05).

### Effect of CSFs on ROS production

**Figure 1:**
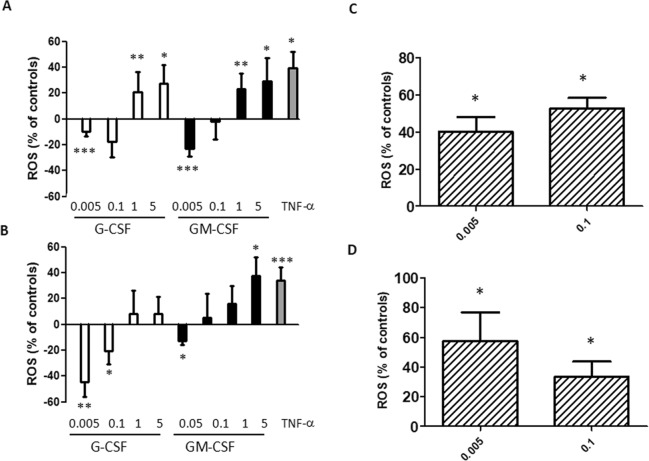
Effect of G-CSF and GM-CSF on oxidative burst. Neutrophils were treated with single (**A**) or both CSFs (**C**) for 50 min, or treated with single (**B**) or both (**D**) CSFs and further incubated with 1 μM fMLP for 5 min. ROS production is given as percent of untreated cells (**A**,**C**), or cells treated with fMLP (**B**,**D**). Concentrations of CSFs are in ng/ml. TNF-α (5 ng/ml) was used as positive control. Results are shown as mean ± SEM of six experiments. Kruskall-Wallis (with Dunn’s test as post hoc test) and Mann-Whitney U-test: *P < 0.05; **P < 0.01; ***P < 0.0001 as compared with untreated controls (**A**,**C**) or fMLP-treated samples (**B**,**D**).

In order to understand whether CSF levels observed in sera from CF patients can modify neutrophil functions, we tested the effect of CSF serum concentrations found in CF patients (0.005–0.1 ng/ml) on the ROS production by circulating neutrophils obtained from non-CF individuals. Neutrophils were incubated with different concentrations of either CSF followed, in some experiments, by fMLP stimulation. When neutrophils were incubated with either G-CSF or GM-CSF only (Fig. 1A), a bimodal effect on the oxidative burst was noticed. At very low concentrations (0.005 ng/ml) the oxidative burst was significantly inhibited, and the decrement was still observed for 0.1 ng/ml but not significantly. We also tested higher concentrations of CSFs (1–5 ng/ml), finding conversely a significant increase in ROS production (Fig. 1A). In priming experiments, *i*.*e*. neutrophils incubated firstly with either CSF and then with fMLP, a similar bimodal behavior was found although with some differences (Fig. 1B). G-CSF did not give a significant enhancing effect at the highest concentrations, while GM-CSF did exert this effect only at 5 ng/ml. Since both cytokines are present in serum, we investigated the combined effect of G-CSF and GM-CSF on the respiratory burst. Thus, both cytokines at low concentrations (0.005 and 0.1 ng/ml) were tested as either direct stimulators or primers of ROS production. As shown in Fig. 1C, the contemporary presence of both cytokines at 0.005 or 0.1 ng/ml exerted a stimulating effect on ROS levels, an effect confirmed in priming experiments (Fig. 1D). Overall, these data indicate that the presence of both CSFs determines a stimulation of neutrophil oxidative burst at the lowest serum concentrations found in CF patients (0.005–0.1 ng/ml), also when neutrophils are primed by cytokines and then stimulated by bacterial products.

### Effect of CSFs on degranulation

**Figure 2:**
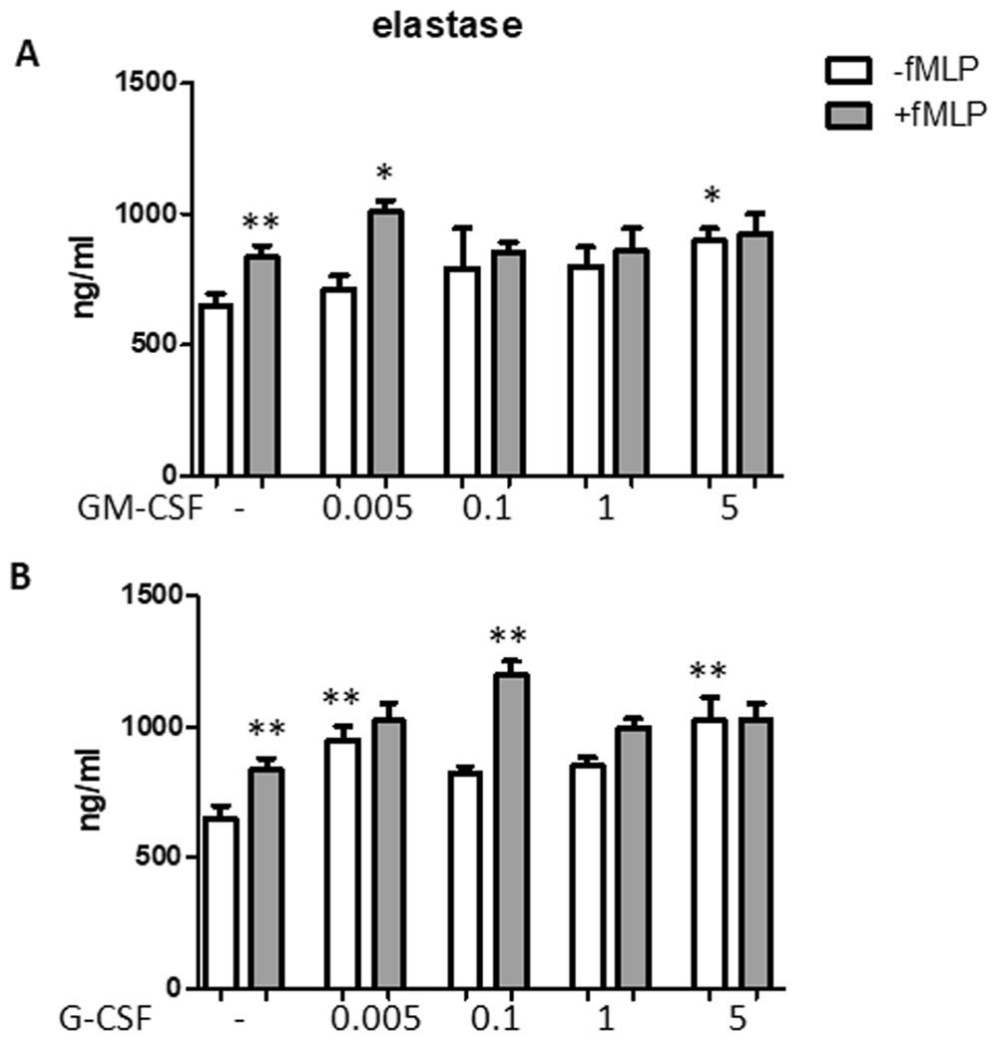
Effect of GM-CSF and G-CSF on elastase secretion. Neutrophils were incubated with either GM-CSF (**A**) or G-CSF (**B**) at the concentration indicated for 50 min in the absence (white columns) or presence (grey columns) of 1 μM fMLP. fMLP was used as positive control. Elastase levels were measured in the supernatants. Results are shown as mean ± SEM of five experiments. ANOVA (with Tukey’s post hoc test) and Student t-test. In both panels, **P < 0.01: +fMLP controls vs. -fMLP controls; *P < 0.05 and **P < 0.01 as compared with untreated and –fMLP controls or untreated and +fMLP controls.

To investigate degranulation, we analyzed elastase as a marker of primary granules (Fig. 2). fMLP increased significantly elastase secretion (P < 0.01). Elastase levels were increased by G-CSF at 0.005 and 5 ng/ml in the absence of fMLP (both P < 0.01 as compared with untreated cells), while only 0.1 ng/ml increased fMLP-mediated elastase levels over those elicited by fMLP (P < 0.01). GM-CSF exerted a stimulatory effect at 5 ng/ml when used as direct stimulus (P < 0.05) and at 0.005 ng/ml when used to prime fMLP-mediated response (P < 0.05).

**Figure 3:**
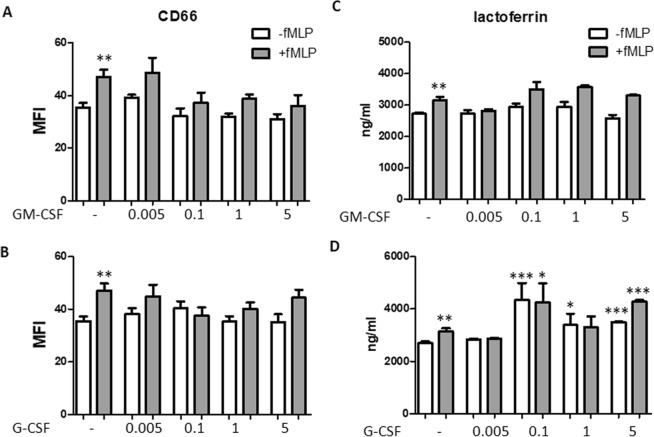
Effect of GM-CSF and G-CSF on CD66b expression and lactoferrin secretion. Neutrophils were incubated with either GM-CSF (**A**,**C**) or G-CSF (**B**,**D**) at the concentration indicated for 50 min in the absence (white columns) or presence (grey colums) of 1 μM fMLP. fMLP was used as positive control. CD66b levels (**A**,**B**) were analyzed by cytofluorimetry and are expressed as MFI. Lactoferrin levels (**C**,**D**) were measured in the supernatants. Results are shown as mean ± SEM of five experiments. ANOVA (with Tukey’s post hoc test) and Student t-test. In all four panels, **P < 0.01: + fMLP controls vs. -fMLP controls; *P < 0.05, **P < 0.01, and ***P < 0.0001 as compared with untreated and –fMLP controls or untreated and + fMLP controls.

As markers of secondary (or specific) granules, we studied CD66b and lactoferrin. CD66b is a receptor whose levels are increased during exocytosis of specific granules^25^. Although fMLP mobilized CD66 on neutrophils plasmamembrane (P < 0.01 as compared with untreated cells), both G-CSF and GM-CSF did not modify the CD66b expression significantly, either in the absence or in the presence of fMLP (Fig. 3). Lactoferrin secretion was significantly enhanced when neutrophils were stimulated with fMLP (P < 0.01). Whereas G-CSF was able to increase lactoferrin levels at the concentrations of 0.1, 1, and 5 ng/ml in the absence of fMLP (P < 0.0001, P < 0.05, and P < 0.0001, respectively), and at 0.1 and 5 ng/ml in the presence of fMLP (P < 0.05 and P < 0.0001, respectively), no effect was observed with GM-CSF (Fig. 3).

**Figure 4:**
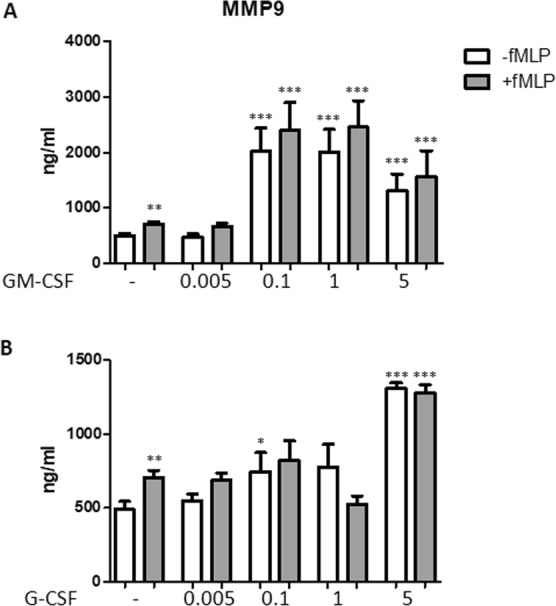
Effect of GM-CSF and G-CSF on MMP-9 secretion. Neutrophils were incubated with either GM-CSF (**A**) or G-CSF (**B**) at the concentration indicated for 50 min in the absence (white columns) or presence (grey columns) of 1 μM fMLP. fMLP was used as positive control. MMP-9 levels were measured in the supernatants. Results are shown as mean ± SEM of five experiments. ANOVA (with Tukey’s post hoc test) and Student t-test. In both panels, **P < 0.01: +fMLP controls vs. −fMLP controls; *P < 0.05, **P < 0.01, and ***P < 0.0001 as compared with untreated and −fMLP controls or untreated and +fMLP controls.

To study tertiary granules, we measured the levels of MMP-9 in the supernatants of neutrophils (Fig. 4). fMLP increased MMP-9 secretion significantly (P < 0.01). GM-CSF determined a significant positive secretion on MMP-9 between 0.1 and 5 ng/ml, either with or without fMLP stimulation (all P < 0.0001 as compared with respective controls). G-CSF increased MMP-9 secretion significantly at the concentrations of 0.1 and 5 ng/ml in the absence of fMLP (P < 0.05 and P < 0.0001, respectively), while this cytokine increased fMLP-dependent MMP-9 secretion only at the highest concentration of 5 ng/ml (P < 0.0001).

### Effect of CSFs on chemotaxis

**Figure 5:**
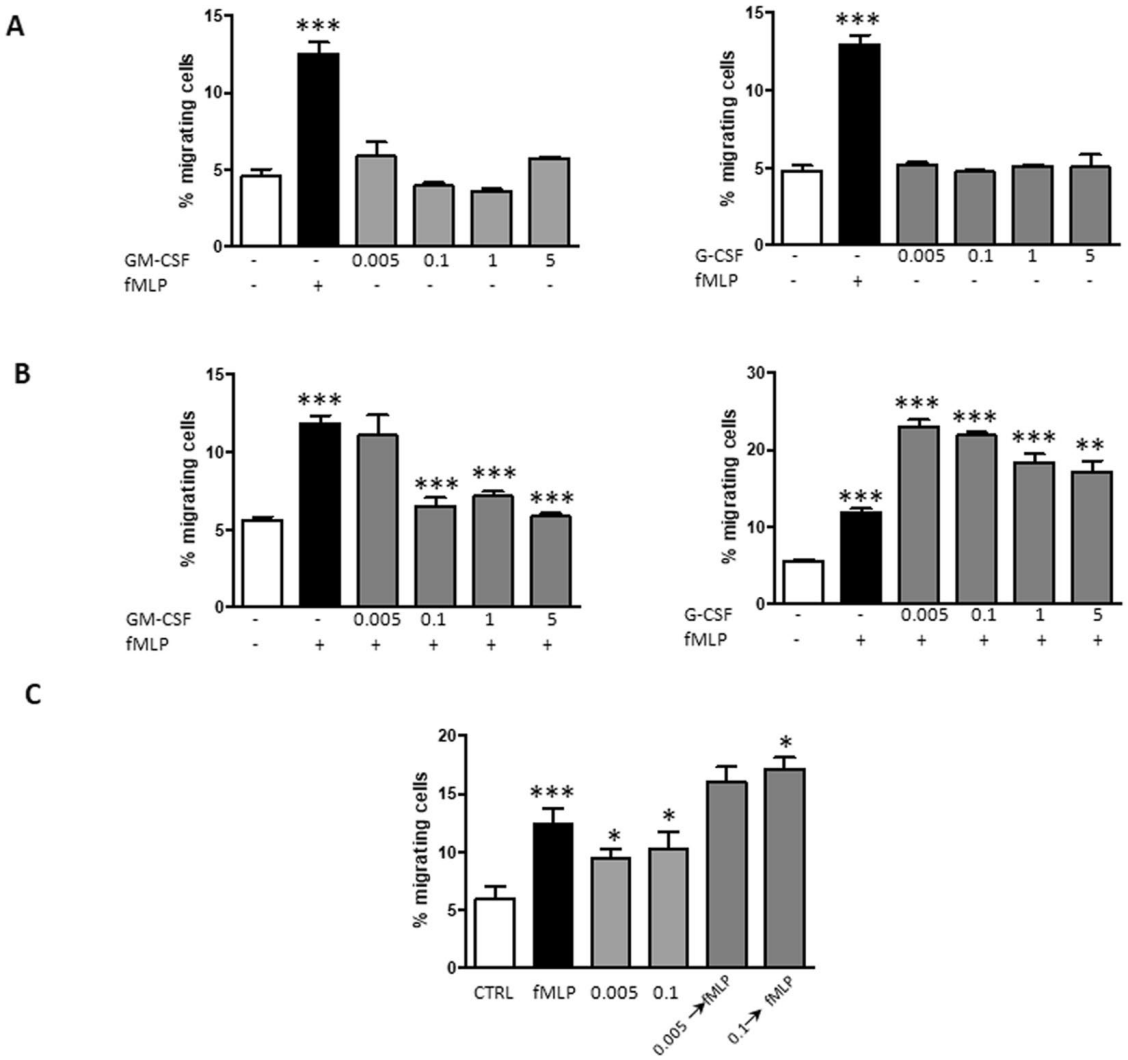
Effect of GM-CSF and G-CSF on neutrophil chemotaxis. (**A**) Neutrophils were allowed to migrate for 2.5 h in the absence or presence of either GM-CSF or G-CSF at the indicated concentrations. fMLP (1 μM) was used a positive control. (**B**) Neutrophils were pre-treated with either CSF for 50 min and allowed to migrate along a fMLP gradient for 2.5 h. (**C**) Neutrophils were either allowed to migrate for 2.5 h in the absence or presence of both CSFs contemporarily presented in the lower chamber at low concentrations (0.005 or 0.1 ng/ml) and with fMLP as positive control, or first pre-treated with both CSFs contemporarily for 50 min at the same concentrations and then allowed to migrate along a fMLP gradient for 2.5 h. Data are shown as percentage of migrated neutrophils as compared with total neutrophils added in the apical compartment. Results are shown as mean ± SEM of six experiments. ANOVA (with Tukey’s post hoc test) and Student t-test. In all panels, ***P < 0.0001: +fMLP controls vs. -fMLP controls; *P < 0.05, **P < 0.01, and ***P < 0.0001 as compared with untreated and –fMLP controls or untreated and +fMLP controls.

CSFs were tested for induction of chemotaxis in a Transwell system. As shown in Fig. 5A, fMLP increased neutrophils chemotaxis significantly (P < 0.0001), while both GM-CSF and G-CSF had no direct chemotactic activity on neutrophils. In order to unravel a “priming” effect, neutrophils were first incubated with either CSFs for 50 min and then allowed to migrate along a fMLP gradient. Pre-incubation of neutrophils with GM-CSF at all the concentrations determined a significant reduction of chemotaxis as compared with fMLP only (Fig. 5B). On the contrary, neutrophils that were pre-activated with G-CSF showed an increase in chemotaxis at all concentrations (Fig. 5B).

Chemotaxis was evaluated when neutrophils were incubated with both cytokines at the same time and at the lowest concentrations, either when used as direct agonists or primers of fMLP effect (Fig. 5C). Interestingly, a heightened effect on chemotaxis were observed when both cytokines were used as direct agonists as compared when used as a single agent (P < 0.05 for both 0.005 and 0.1 ng/ml). When used as priming agents, the chemotactic response was increased over that induced by fMLP but the effect was an average of that determined by single cytokines (Fig. 5C). However, significance was obtained only when neutrophils were primed with 0.1 ng/ml of both cytokines (P < 0.05).

## Discussion

Previously, GM-CSF and G-CSF levels were measured in serum of CF patients in stable condition and their ratio identified patients with chronic *P*. *aeruginosa* infection versus those with no chronic infection^17,18^. Although a direct comparison of results obtained in these studies with ours cannot be made, since almost all patients were chronically infected with *S*. *aureus*, *P*. *aeruginosa* or *B*. *cepacia*, CSFs levels in CF patients reported in this study are quite in good agreement with those previously found. In sputum, G-CSF concentrations were higher than GM-CSF ones (Table 2). Few others have reported higher levels of G-CSF then GM-CSF in sputum^13,19^, although not with big differences as reported herein by us. These authors dosed G-CSF and GM-CSF in sputum of CF patients without any addition of anti-proteases, as we did in this paper. Although it is conceivable that sputum elastolytic activity may cleave CSFs as it happens with other well-known soluble and cellular substrates^26^, in-depth future studies are needed to address this issue. NETs could either enhance CSF degradation, due to their protease content^27^, or protect from proteolytic degradation, indicating that differences in sputum stability of GM-CSF and G-CSF should be carried out in the presence of NETs.

G-CSF production is primarily confined to monocytes/macrophages, fibroblasts, and endothelial cells and is enhanced in response to bacterial products and inflammatory mediators^28^, such as TNF-α, a cytokine which is highly produced in CF lungs particularly during pulmonary exacerbations^10^. G-CSF expression is often induced during infections, resulting in high levels both systemically (*i*.*e*, in blood) and locally in inflammatory fluids in both mice^29,30^ and in humans^31–33^. On the other hand, GM-CSF is normally produced locally by the alveolar macrophages^34^, being the first line of defense when clearing bacteria^35^. It appears that its main functions are to regulate surfactant homeostasis and primarily macrophage activity and differentiation. Our data suggest that chronic infection of CF lungs stimulates the production of G-CSF rather than of GM-CSF.

G-CSF and GM-CSF are chemoattractants for neutrophils and could prime these cells for an enhanced respiratory burst activity after stimulation with several agents. Moreover, both cytokines enhanced the neutrophil  antibody-dependent cellular cytotoxicity (ADCC), adherence, phagocytosis and killing of microorganisms^36^. G-CSF and GM-CSF bind to their cognate cell surface receptors on neutrophils, *i*.*e*. G-CSFR (encoded by *CSF3R*) and GM-CSFR (encoded by *CSF2R*). While G-CSFR is a homodimeric receptor, GM-CSFR is a heterodimer comprising the cytokine-specific α subunit and the βc subunit common also to IL-3 and IL-5 receptors. Both G-CSF and GM-CSF are type I receptors that lack intrinsic catalytic activity but trigger the activation of the JAK-STAT pathway, SRC family kinases, and the PI3K and MAPK pathways^37,38^. The β-chain-containing GM-CSF and the homodimeric G-CSF receptors utilize STAT2 and STAT5 or STAT3, but it is unclear how the specificity of the receptor is carried over through different JAK and STAT family members and how a limited number of JAK and STAT components are able to trigger specific signals^39^.

This complexity is reflected by the results obtained in this paper. Indeed, we show that both cytokines, tested as single stimulus, had similar effects on ROS production but exerted different modifications of degranulation and chemotaxis. To make this outcome even more complex, when the two CSFs were present at the same time as direct agonists or priming agents for fMLP, the results showed that they increased ROS production by an effect that has to be determined yet, possibly given by an overlapping of the signaling pathways.

In the case of chemotaxis, previous studies had reported divergent results, some demonstrating G-CSF and GM-CSF as chemotactic for neutrophils^40–42^, others showing that they were negative factors for chemotaxis^43,44^. The timing of exposure and the chemotactic agent to which CSFs prime neutrophils are likely responsible for these different results. It is worth noting that all these studies were carried out by using single CSFs as chemotactic agents, and indeed we obtained similar differing results under these conditions. However, studying chemotaxis with both cytokines present contemporarily, which has not been done before to the best of our knowledge, showed that they could exert a positive effect on neutrophils chemotaxis, either as direct agonists or when used as priming agents.

Once attracted into the airways, CSFs would assist in maintaining the neutrophils at the site of inflammation, promoting their survival^45,46^, and inducing superoxide production where it is required most. Specifically, at the lowest concentrations found in CF patients (0.005–0.1 ng/ml), CSFs exerted a negative effect on the respiratory burst, whereas higher concentrations increased ROS levels. Interestingly, a signaling switch for GM-CSFR has been observed. At concentration as low as 1 pM (0.014 ng/ml), GM-CSF mediates βc Ser^585^ phosphorylation, leading to 14–3–3 binding, PI3K activation, and hematopoietic cell survival, whereas at concentrations of 10 pM (0.14 ng/ml) or more, GM-CSF mediates βc Tyr^577^ phosphorylation, Shc recruitment, and PI3K activation, thereby promoting both survival and proliferation^47^. However, under our experimental conditions, the incubation of neutrophils with both cytokines at low concentrations enhanced ROS production, which may simulate higher concentrations. Whatever the experimental set-up, the two cytokines exerted their effect directly, and the magnitude of ROS levels was similar to that obtained with priming experiments, suggesting that priming for fMLP stimulation is not important with low CSF concentrations on blood neutrophils. Whether this phenomenon occurs also in sputum neutrophils has to be ascertained, but we can anticipate that since the concentration of bacterial stimuli should be higher in the local lung environment, the already stimulated neutrophils should react more to these agonists. Together with the combined effect of local CSFs, neutrophils should be more active and secrete more ROS than in the blood. While this would increase the bactericidal activity of neutrophils during non-CF bacterial infections, however, this would create more tissue damage than benefits in the CF lung, since opportunistic CF bacteria, such as *P*. *aeruginosa*, are protected by alginate exopolysaccharides. Moreover, GM-CSF primes neutrophils for NET formation by other stimuli, such as LPS or C5a, and ROS are implied in their formation^21^. One caveat to these considerations is that sputum cytokines levels, including CSFs, reflect heterogeneity of airway inflammation across lung regions^48^ and also bacterial-derived products are present in sputum as a consequence of the complex CF lung environment^49^. Thus, the sputum CSF levels are likely to be an average of higher and lower local G-CSF/GM-CSF concentrations in CF lungs, implying that our results should be confirmed *in vivo* by exploring different lung regions (e.g. upper and lower lobes). Finally, since NETs have been correlated with CF lung disease progression, it would be interesting to study NETs in blood neutrophils upon CSF stimulation. Since we could appreciate the secretion of granule enzymes in our assays with differences between GM-CSF and G-CSF stimulation, and NETs or partially cleaved NET fragments can sequester active proteases^27^, the evaluation of NETs, with both lower and higher concentrations of both CSFs present contemporarily, will allow to obtain crucial information on neutrophils’ activation already in the blood and avoid the issue of local heterogeneity. This brings the attention to blood neutrophils as possible non-invasive markers of CF lung disease.

As for the degranulation, we found that G-CSF and GM-CSF were able to induce elastase and MMP-9 release by neutrophils, at concentrations as low as 0.005–0.1 ng/ml, while lactoferrin secretion was enhanced significantly only by G-CSF. As pointed out above, differences in signal transduction pathways may generate different responses, however this issue has been studied more deeply in hematopoietic progenitors and less in mature neutrophils, and should be thoroughly investigated by means of pathway inhibitors or in neutrophils bearing mutations inactivating these pathways.

The direct and priming effects on elastase and MMP-9 shown by neutrophils upon stimulation with both CSFs might be important for the pathogenesis of matrix breakdown and remodeling occurring in infected CF bronchioles leading to bronchiectasis^50^. Neutrophil elastase has been recognized as a key risk factor for the onset and early progression of CF lung disease^51,52^. The role of elastase in the pathogenesis of CF lung disease is highlighted by the fact that this protease has been implicated in several key features of CF lung disease including airway inflammation, goblet cell metaplasia and mucus hypersecretion, and proteolytic damage of airway walls^53–59^. Airway and serum/plasma MMP-9 protein and activity were shown to be elevated in CF and negatively correlated with the respiratory function and with its decline rate^60–65^. Moreover, plasma MMP-9a is associated with bacterial infection^65^. Although this is not the only MMP found to be elevated in CF airway secretions, these findings highlight its possible role in neutrophil-mediated progression of CF lung diseases.

G-CSF had an enhancing effect on unstimulated and fMLP-induced release of lactoferrin, which is an iron-chelating glycoprotein of innate immunity and represents a major endogenous antimicrobial constituent of airway secretions^66^. Lactoferrin exerts several functions as inhibition of microbial growth, biofilm development, and bacterial invasion of host cells^67,68^. Also due to its demonstrated anti-inflammatory activities, it has been suggested that lactoferrin is an important molecule for interrupting the vicious circle involving iron homeostasis dysregulation, bacterial infection, and inflammation in CF^69^. Thus, lactoferrin increase might be part of a negative feedback mechanism operating when hyperinflammation occurs, as in CF lungs, however other studies are mandatory to validate this hypothesis.

Although the number of replicates in degranulation studies witnesses the robustness of data, conclusive remarks should be made only when the levels of granule enzymes as well as NETosis, which is strictly interrelated, will be analysed following neutrophil stimulation with both cytokines present at the same time, which is the focus of ongoing studies.

All-in-all, our data strongly suggest that circulating CSFs stimulate neutrophils to display a heightened chemotactic phenotype, and to produce more ROS and granule enzymes. Thus, neutrophils once entered into the CF lungs might be more damaging than protecting the airways from the infection. Our data show that G-CSF is highly produced than GM-CSF in the infected lung of CF patients and it might be the factor promoting further neutrophils’ activation and then more damage. Therefore, it could be a target of inhibitory therapy.

In conclusion, CSF levels found in serum of CF patients modify the behavior of circulating neutrophils by modulating ROS production, degranulation of proteases, and chemotaxis. On the basis of these results it might be reasonable to assume that, influenced by these levels, circulating CF neutrophils are already activated before entering CF lungs.

## Patients and Methods

### Patients

In this study, 21 patients in stable condition and 19 patients in acute exacerbation were considered. Exacerbation was defined as a deterioration in symptoms perceived by the patient and included an increase in coughing, sputum production, dyspnea, decline in forced expiratory volume in 1 sec (FEV_1_) compared with previous best, weight loss and fever^70^. Patients in acute exacerbation were studied before and after a 10-day course of antibiotic therapy. The ratios between males and females were 11:10 and 6:13 in stable and acute patients, respectively. Patients age was 30.9 ± 2.2 for stable and 30.0 ± 2.9 for acute (mean ± SEM). Upon admission, all clinical and laboratory parameters were collected: white blood cell count, neutrophil percentage, CRP (mg/dl), FEV_1_ (% predicted for sex and height), and pathogen colonization. The stable patients were colonized, as single or coupled, by *P*. *aeruginosa* (n = 10), *S*. *aureus* (n = 8), *B*. *cepacia* (n = 3), nontuberculous mycobacteria (n = 1) and *Enterobacter* (n = 1), while acute patients presented *P*. *aeruginosa* (n = 10), *S*. *aureus* (n = 4), nontuberculous mycobacteria (n = 5), *E*. *coli* (n = 3), and *B*. *cepacia* (n = 2). Most patients, *i*.*e*. 20 in stable condition (95%) and 17 in acute exacerbation (89%), were chronically infected, as established by international guidelines^71,72^. The study was approved by the ethics committee of the Azienda Ospedaliera Universitaria “Policlinico” of Bari (no. 1373/CE/2012) and performed in accordance with the 2013 amended Declaration of Helsinki. Written informed consent from the adult study subjects or informed consent from a parent and/or legal guardian for study participation of subjects under the age of 18 was obtained.

### Blood and sputum processing

Blood samples were collected and immediately centrifuged for 15 min at 1,590 × g; serum was separated and stored at −80 °C until assays were performed. At the same time, the sputum spontaneously coughed was collected in sterile cups and was processed immediately. Sputum samples (0.5–2 mL) were diluted 1:1–1:6, with phosphate-buffered saline (PBS), immediately vortexed and then centrifuged for 5 min at 7000 × g. Each supernatant was immediately removed and stored in aliquots at −80 °C before assaying.

### Measurement of CSFs levels in sputum and serum

G-CSF and GM-CSF levels in sputum and serum specimens were measured by an ELISA kit (Invitrogen, Corporation, Camarillo, CA, USA). The sensitivity of the Hu GM-CSF kit (cat. #KHC2011) and of the Hu G-CSF kit (cat. #KHC2031) is <3 pg/ml and <20 pg/ml, respectively. Obtained values were normalized according to the initial dilution of the sample.

Some of the samples were below the detection limit of the assay: serum G-CSF (stable = 1; pre-therapy = 1; post-therapy = 2); sputum G-CSF (stable = 1; pre-therapy = 2; post-therapy = 4); serum GM-CSF (post-therapy = 1); sputum GM-CSF (stable = 2; pre-therapy = 1; post-therapy = 1).

### Neutrophils isolation

Neutrophils were obtained from whole blood of healthy human donors. Briefly, neutrophils were isolated in LPS–free conditions by Dextran sedimentation followed by Ficoll-Hypaque density gradient centrifugation. The lower phase containing neutrophils was resuspended in RPMI (Roswell Park Memorial Institute) medium, washed and contaminating erythrocytes were removed by hypotonic lysis. The resulting cell population contained greater than 95% mature neutrophils as assessed by flow cytometric analysis with BD FACScalibur (BD Biosciences, San Jose, CA, USA) using a FITC-conjugated mouse monoclonal antibody against CD16b (Santa Cruz Biotechnology, Santa Cruz, CA, USA), used at 1 μg/1 × 10^6^ cells^25^. An irrelevant mouse FITC-conjugated monoclonal antibody was used as negative control. In total, 10,000 events were recorded per staining. Neutrophil viability was assessed by Trypan blue exclusion assay, with viable neutrophils >98%.

### Neutrophils stimulation

GM-CSF, G-CSF, TNF-α, and N-formylmethionine-leucyl-phenylalanine (fMLP) were purchased from Sigma-Aldrich (Milan, Italy). Neutrophils (3 × 10^6^/mL) were incubated either with G-CSF or GM-CSF or primed with CSFs for 50 min and then stimulated with 1 μM fMLP. For ROS measurement experiments, DMSO was used as a negative control for fMLP and TNF-α (50 ng/ml) was used as positive control for priming. In some experiments, both CSFs were present together at low concentrations (0.005–0.1 ng/ml) both as direct stimulators or priming agents for fMLP.

### Measurement of intracellular ROS

Neutrophils were either unstimulated or stimulated with CSFs, incubated with 2 μM H_2_DCFH-DA (2′,7′-Dichlorodihydrofluorescein diacetate; Invitrogen) for 10 min, washed twice in PBS and exposed to 1 μM fMLP. After 10 min, ROS-mediated oxidation of H_2_DCFH-DA into the highly fluorescent 2′,7′-dichlorofluorescein (DCF) was measured by flow cytometry (BD FACScalibur) within 30 min after termination of stimulation. In total, 10,000 events were recorded per staining. Control neutrophils (*i*.*e*. incubated with DMSO) and neutrophils stimulated with fMLP gave an intracellular fluorescence (MFI = mean fluorescence intensity) of 160.5 ± 14.4 and 207.0 ± 17.1 (mean ± SEM; n = 18; Mann-Whitney U-test: P < 0.05), respectively. MFI obtained with study samples was calculated as % of controls mean (*i*.*e*, DMSO or fMLP).

### Analysis of neutrophil degranulation

Neutrophils (7 × 10^6^ cells/ml) were incubated with G-CSF or GM-CSF (0.005, 0.1, 1 and 5 ng/ml) for 50 min and evaluated either unstimulated or stimulated with 1 μM fMLP for 5 min. Supernatants were snap-frozen at −80 °C. The release of granule proteins (elastase, lactoferrin and MMP-9) were assessed by enzyme-linked immunosorbent assay (R&D System). As an alternative approach to degranulation of secondary granules, the membrane expression of CD66b was investigated. Neutrophils were fixed in 4% (w/v) paraformaldehyde following stimulation and analyzed by flow cytometry (BD FACScalibur) using a fluorescein isothiocyanate (FITC)-labeled mouse anti-human CD66b antibody (Miltenyi Biotec, Bologna, Italy) used according to manufacturer’s instructions. An irrelevant FITC-conjugated mouse IgG1 was used as negative control. In total, 10,000 events were recorded per staining. Data were expressed as MFI.

### Chemotaxis

Neutrophils (1 × 10^6^ per filter) were added in the upper compartment of a Transwell insert (Corning, 3 μm of pore size), while in the lower compartment, 1 μM fMLP, GM-CSF or G-CSF was added. After  2.5 h, neutrophils that crossed the insert were recovered in the lower compartment and quantified by flow cytometry (BD FACScalibur), and data were expressed as percentage of total neutrophils added in the upper compartment. In some experiments, neutrophils were pre-incubated with either CSF for 50 min, added to the upper compartment and allowed to migrate along a fMLP gradient for 2.5 h.

### Statistical analysis

Since data presented either normal or non normal distribution of values (Shapiro-Wilk), the differences were computed by parametric and non parametric tests, respectively. Clinical parameters, CSFs levels in serum and sputum, and ROS levels showed a non normal distribution, thus Kruskall-Wallis analysis of variance (with Dunn’s test as post hoc test) and Mann-Whitney U-test for pairwise comparisons were used. Degranulation and chemotaxis data presented a normal distribution, hence we used ANOVA with Tukey’s post hoc test and Student t-test for pairwise comparisons. Correlation analysis was carried out by computing Spearman’s rank correlation coefficients. The difference was considered significant when P < 0.05. The analysis was carried out using the software Prism 4 (GraphPad Software, Inc., La Jolla, CA, USA).

## Supplementary information

Supplementary Material

## Data Availability

All data generated or analysed during this study are included in this published article.
