# Supplementary material for: G-CSF and GM-CSF Modify Neutrophil Functions at Concentrations found in Cystic Fibrosis
Source: Sci Rep. 2019 Sep 10;9:12937. doi: 10.1038/s41598-019-49419-z (PMC6736848; doi:10.1038/s41598-019-49419-z)
Supplement: Supplementary file 1 — Supplementary Material [file 41598_2019_49419_MOESM1_ESM.pdf]

G-CSF AND GM-CSF MODIFY NEUTROPHIL FUNCTIONS AT CONCENTRATIONS FOUND IN CYSTIC FIBROSIS

Stefano Castellani[1§], Susanna D'Oria[2§], Anna Diana[3], Angela Maria Polizzi[3], Sante Di Gioia[1], Maria Addolorata Mariggiò[2], Lorenzo Guerra[4], Maria Favia[4], Angela Vinella[2], Giuseppina Leonetti[5], Domenica De Venuto[5], Crescenzio Gallo[6], Pasqualina Montemurro[2*], Massimo Conese[1*]

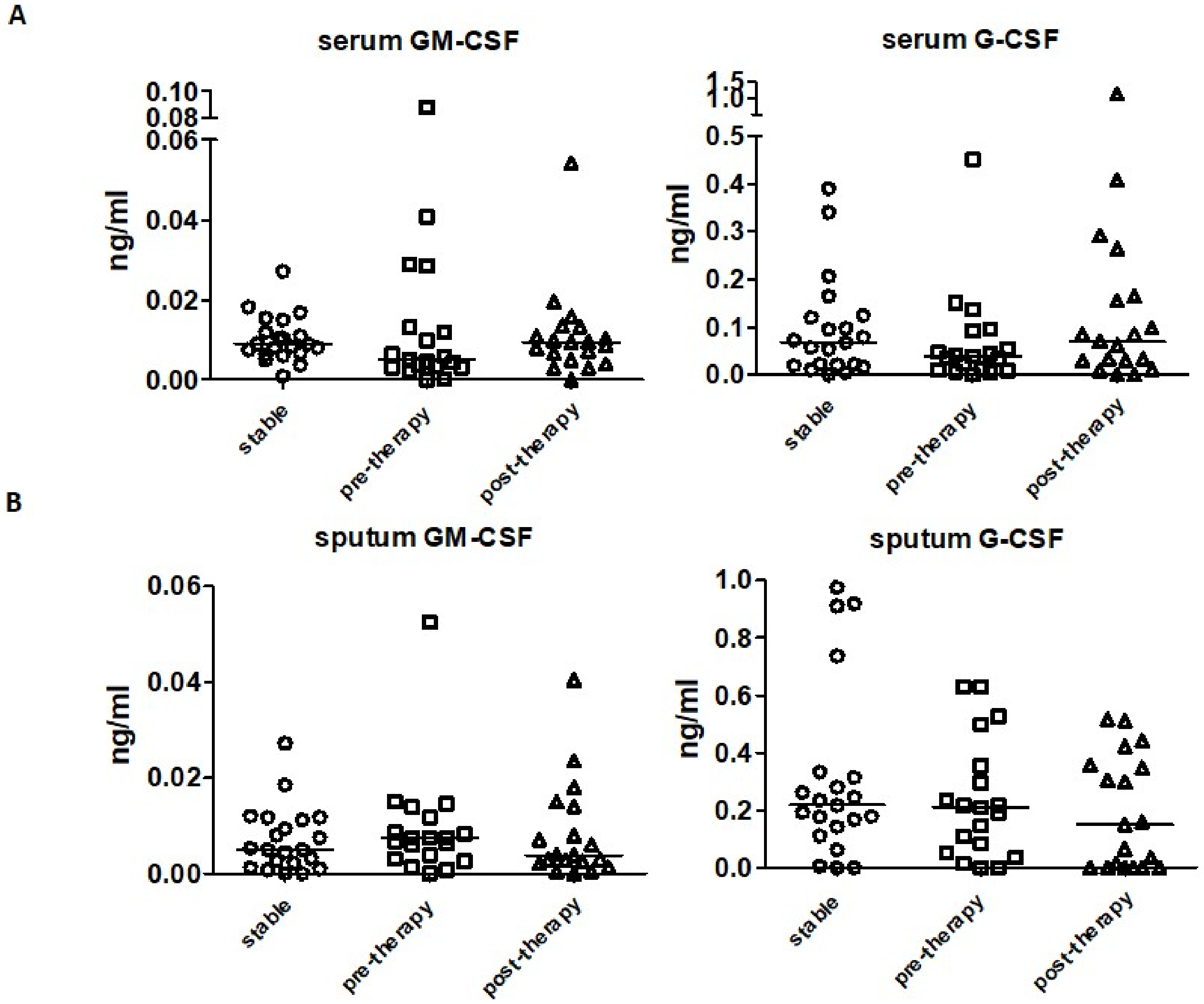

Supplementary Fig. 1 Serum (A) and sputum (B) levels of GM-CSF and G-CSF in CF patients in stable conditions (n=21) and CF patients in acute exacerbation (n=19) before (pre) and after (post) a course of antibiotic therapy. The median is represented as a solid line.
